# Tuina for functional constipation: A protocol for the systematic review of randomized clinical trials

**DOI:** 10.1097/MD.0000000000014775

**Published:** 2019-03-08

**Authors:** Congan Wang, Xinghe Zhang, Dandan Wang, Bin Shi, Guodong Sun, Bo Zhang, Liang Zou

**Affiliations:** a School of Acupuncture-Tuina, Shandong University of Traditional Chinese Medicine; b Affiliated Hospital of Shandong Academy of Medical Sciences; c Shandong Medicinal Biotechnology Centre, Jinan, Shandong Province, China.

**Keywords:** functional constipation, massage, protocol, systematic review, Tuina

## Abstract

**Background:**

Functional constipation (FC) is one of the most common functional gastrointestinal disorders, which brings many negative impacts to the patient's daily life. In the treatment of FC, Tuina (Chinese massage) is often performed as complementary and alternative medicine and shows a good effect in many clinical trials. However, no high-quality systematic review was taken to show the efficacy and safety of Tuina for FC.

**Methods:**

The electronic databases of Cochrane Library, Web of Science (WOS), MEDLINE, Wiley, Springer, EMBASE, Chinese Science Citation Database, China National Knowledge Infrastructure, Chinese Biomedical Literature Database, Chinese Scientific Journal Database, Wan-fang database, and other databases will be searched from the establishment to February 1, 2019. Randomized controlled trials about this theme will be retrieved. Independent reviewers will operate literature retrieval, duplication removing, screening, quality evaluation, data analyses by EndNote (X9) and Review Manager (5.3.5). Meta-analysis, subgroup analysis and/or descriptive analysis will be performed based on the included data form.

**Results:**

Evidenced outcome will be provided from defecation frequency, stool consistency (Bistol stool scale), success rates, quality of life, proportion of patients using laxatives, and adverse effects.

**Conclusion:**

This review will provide evidence of whether Tuina is an effective and safe intervention for FC.

**Trail registration number:**

This protocol of systematic review has been registered on PROSPERO website (No. CRD42019119722).

## 1 Introduction

### 1.1 Description of the condition

Functional constipation (FC) is a functional gastrointestinal disorder in which symptoms of difficult, infrequent, incomplete defecation predominate but does not meet irritable bowel syndrome criteria.^[1–3]^ According to the survey report, the prevalence of FC was 15% in North America, 17.1% in Europe, 15.3% in Oceania, and14% in Asia.^[4–6]^ As a common disease both in children and adults, FC severely influence the quality of patients’ daily life and consumes many resources.^[7–10]^

### 1.2 Description and function of intervention

Tuina (Chinese massage) is an ancient physical therapy of traditional Chinese medicine (TCM) which can be traced back to around 220 BC. Based on different meridian-acupoint theory and operation method, pediatric Tuina for children was separated from Tuina for adults in the Ming Dynasty.^[11,12]^ Up to now, many universities of TCM offer the subjects of Tuina and pediatric Tuina for medicos.^[13]^ In many textbooks and clinical trials, teachers and doctors explained how to treat FC by Tuina, such as rubbing abdomen, pressing Tianshu (St25), pressing Dachangshu (BL25), pushing Qijiegu.^[14–20]^

### 1.3 Why this systematic review is needed

FC increases the risk of hemorrhoids, rectal prolapse, cardiovascular disease, tristimania, and even intestinal cancer.^[8,10,21]^ In clinical, pharmacologic agents (laxatives and fiber supplements) and non-pharmacologic therapies (exercise increasing, fluid intake, and bowel habit training) were usually recommended for FC. But for the curative effect, these measures are still limited and unclear.^[22,23]^ As external physiotherapy for FC, Tuina was widely used in clinical.^[14–18]^ However, the evidence was still limited based on subjectivity judgment, nonstandard measurement, and other factors. On the other hand, no related evidenced review or protocol was published. So, this review is urgently needed.

## 2 Methods

This review has been registered on PROSPERO register of systematic reviews network (No. CRD42019119722). All steps of this systematic review will be performed according to the Cochrane Handbook (5.2.0).

### 2.1 Selection criteria

#### 2.1.1 Types of literature

As the randomized controlled trials (RCTs) is reliable and feasible, RCTs will be included only. After the presearch, published clinical trials which reported the efficacy and safety on Tuina for FC will be included. RCTs that involve at least 1 Tuina treatment and 1 control treatment (or blank treatment) will be included. Literature of animal research, case report, review, and meta-analysis will be excluded.

#### 2.1.2 Types of patients

Patients who were diagnosed as FC according to ROME II, III, or IV criteria will be included, without limits on gender, age, race, nationality, and medical units.

#### 2.1.3 Types of interventions and comparisons

Interventions of multiple Tuina therapies (eg, pressing, pushing, rubbing, transiting, and stroking) and comparisons of other treatments will be included. Interventions of Tuina combined with other treatments will also be included, only if the other treatments were used as comparisons. Interventions of Tuina and comparisons of other Tuina therapies will be excluded.

#### 2.1.4 Types of outcomes

Main outcomes of this review include defecation frequency and stool consistency (Bistol stool scale). Additional outcomes of this review include treatment success rates, quality of life, proportion of patients using laxatives, and adverse effects.

### 2.2 Electronic search strategy

The electronic databases of Cochrane Library, Web of Science (WOS), MEDLINE, Wiley, Springer, EMBASE, Chinese Science Citation Database, China National Knowledge Infrastructure, Chinese Biomedical Literature Database, Chinese Scientific Journal Database, Wan-fang database, and other databases will be searched from the establishment to February 1, 2019.

Exemplary search strategy of WOS is listed in Table 1 and search terms are conform to medical subject heading. According to the different retrieval modes, keywords may combined with free words and appropriate search mode will be performed.

**Table 1:**
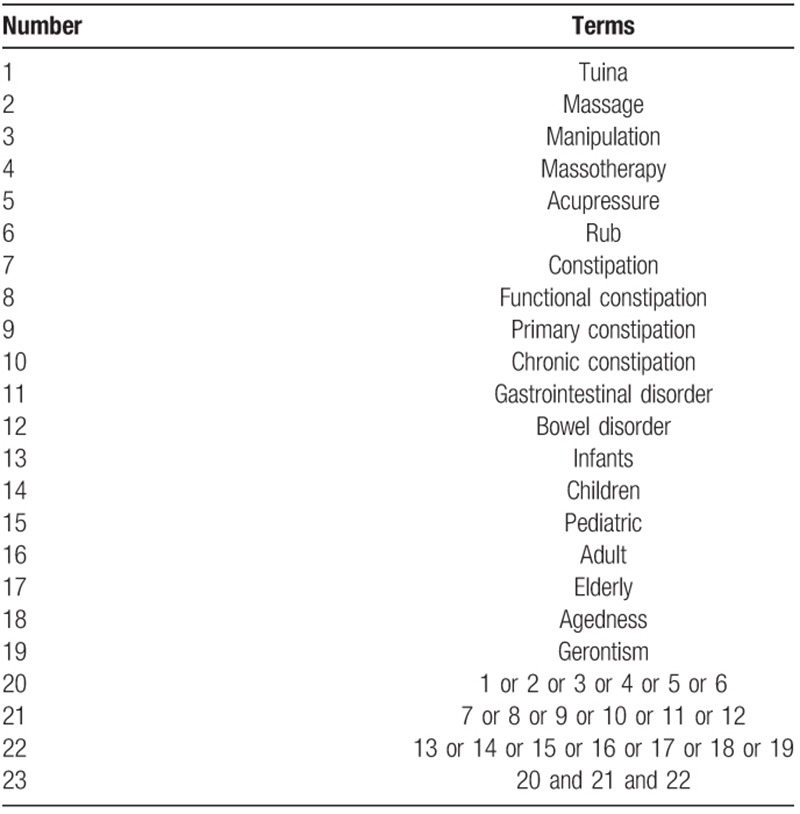
Web of science search strategy.

### 2.3 Data collection and analysis

#### 2.3.1 Selection of literature

**Figure 1:**
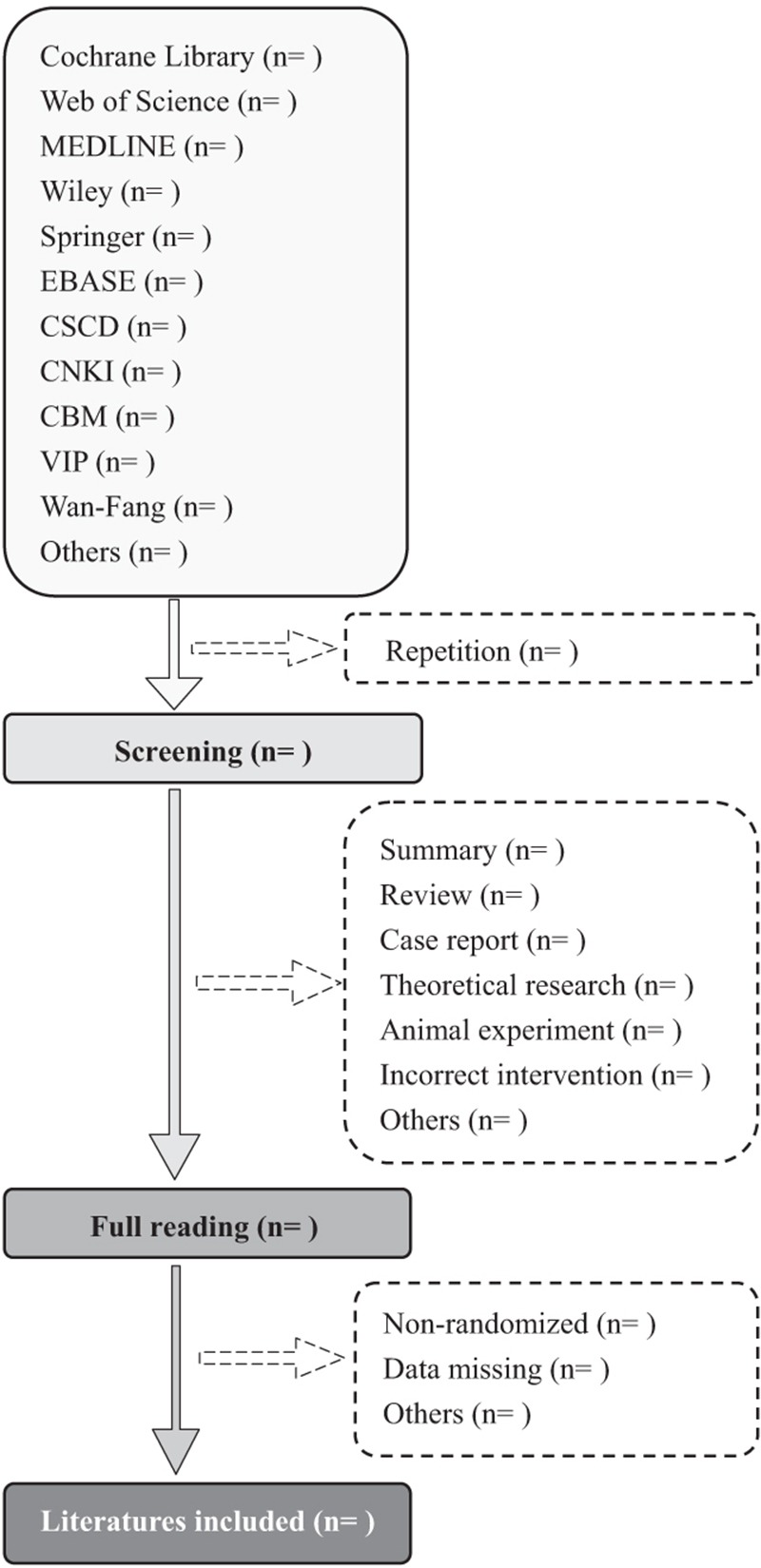
Flow diagram of literature retrieval. CBM = Chinese biomedical literature database, CNKI = China National knowledge infrastructure, CSCD = Chinese science citation database, VIP = Chinese scientific journal database.

Two independent authors (CAW and DDW) will select clinical trials according to the inclusion criteria. After the screening of title and abstract, repetitive, irrelevant, and do not meet the criteria not related literatures will be excluded by EndNote (X9). Screening operation will be performed as Figure 1. We will try to contact the corresponding author when full literatures or requisite data unavailable. Third-party experts will be consulted to determine the selection divergence.

#### 2.3.2 Quality assessment of included literature

Two independent authors (XHZ and BZ) will evaluate the quality of included literature, risk of bias will also be evaluated by Review Manager (5.3.5) according to Cochrane Handbook (5.2.0). Randomized method, allocation concealment, blinding methods, completeness of outcome data, and selective reporting will be assessed. Third-party experts will be consulted when the assessment is in dispute.

#### 2.3.3 Data extraction

Two independent authors (XHZ and LZ) will extract data after selection and quality assessment. The basic information and outcome data of included literature will be extracted, and the details will be compiled to normative form: title (serial number, name of first author, journal source, and published date), participants (age, gender, and number), intervention of intervention and comparison (method, time, and cycle), main outcomes, and additional outcomes.

#### 2.3.4 Measures of treatment effect

Two independent authors (CAW and XHZ) will analyze the treatment effect by Review Manager (5.3.5). Risk ratio (RR) with 95% confidence interval (CI) will be adopted when dichotomous data existence. While the mean difference or standard mean difference with 95% CI will be adopted when continuous data existence. RR form will be changed to analyze when binary data existence.

#### 2.3.5 Dealing with missing data

As the requisite data may be missing in the literature, the author (DDW) will contact the corresponding authors by available contact ways. If the data is still unattainable, random missing will be supposed when analysis the available data.

#### 2.3.6 Assessment of heterogeneity

The heterogeneity of the data will be assessed by *Q*-test and *I*^2^ statistic. The heterogeneity will be deemed as low when *I*^2^ < 50%, moderate (50–75%), high (*I*^2^ > 75%).

#### 2.3.7 Assessment of reporting bias

Funnel plots will be created to assess the reporting bias. Dissymmetry funnel plot indicates high risk of reporting bias, while symmetric funnel plot indicates low risk.

#### 2.3.8 Data synthesis

Meta-analysis, subgroup or descriptive analysis will be performed based on participants’ age, pathogenic factors, intervention methods, measurement methods, and heterogeneity levels, and so on. Fixed-effect model with merger analysis will be applied when heterogeneity is low. Random-effects model with merger analysis will be applied when heterogeneity is moderate. While the heterogeneity is significantly high, subgroup analysis or descriptive analysis will be performed.

#### 2.3.9 Subgroup analysis

Subgroup analysis will be performed when the preliminary result of data synthesis indicates the subgroup is needed. For instance, if the heterogeneity is caused by particular features (eg, participants’ age, pathogenic factors, disease duration, intervention time, intervention cycle, pediatric/adult Tuina, measurement methods), subgroup analysis will be conducted relevant to these categories.

## 3 Discussion

Tuina is external physiotherapy with a long history, which is used for many diseases. As an important treatment with significant effects and cost-effective, Tuina is widely used for FC and the amount of related literature were published. But for the preliminary retrieval and analysis, high-quality trails is still insufficient. Compelling evidence is still unobtainable, valid guide is also difficult to achieve. If necessary trails are still lacking within 2 years, this systematic review will be formally launched and update will be performed every 4 years.

## Author contributions

**Conceptualization:** Congan Wang, Bin Shi.

**Data curation:** Congan Wang, Xinghe Zhang, Dandan Wang, Bo Zhang, Liang Zou.

**Investigation:** Guodong Sun.

**Methodology:** Congan Wang, Xinghe Zhang.

**Methodology:** Congan Wang, Xinghe Zhang.

**Validation:** Bin Shi.

**Visualization:** Congan Wang.

**Writing – original draft:** Congan Wang, Xinghe Zhang.

**Writing – review and editing:** Bin Shi, Guodong Sun.
